# Growth Mixture Modeling of Patient-reported Outcomes After Total Knee Arthroplasty: No Recovery Trajectory Shows Postoperative Decline or Stagnation

**DOI:** 10.5435/JAAOSGlobal-D-25-00107

**Published:** 2025-06-11

**Authors:** Kareem Omran, Colleen Wixted, Daniel Waren, Joshua C. Rozell, Ran Schwarzkopf

**Affiliations:** From the Department of Public Health and Primary Care, University of Cambridge, Cambridge, United Kingdom (Mr. Omran), and the Department of Orthopedic Surgery, NYU Langone Health, New York, NY (Dr. Wixted, Waren, Dr. Rozell, and Dr. Schwarzkopf).

## Abstract

**Background:**

Recovery after total knee arthroplasty (TKA) shows considerable variability in both pain relief and functional improvement. The Knee Injury and Osteoarthritis Outcome Score (KOOS-JR) is a widely used measure for evaluating these outcomes. This study aimed to identify distinct latent recovery trajectories, which represent underlying, unobserved patterns of postoperative recovery inferred from KOOS-JR scores, and to explore patient characteristics associated with these trajectories.

**Methods:**

This retrospective cohort study analyzed patients who underwent primary TKA for osteoarthritis at a tertiary academic center from January 2020 to March 2023. Inclusion criteria required patients to have completed a preoperative KOOS-JR questionnaire and at least two postoperative follow-ups at 1, 3, 6, or 12 months. Exclusion criteria included bilateral or revision procedures. Collected characteristics included age, sex, Body Mass Index, American Society of Anesthesiologists physical status classification, race, smoking status, procedure type, anesthesia type, length of hospital stay, and discharge disposition. Growth mixture modeling was used to model recovery trajectories, with associations evaluated using the “three-step approach.” Model fit was assessed using the Akaike and Bayesian Information Criteria, Vuong-Lo-Mendell-Rubin likelihood ratio, posterior probabilities, and entropy values.

**Results:**

Of 700 eligible patients, growth mixture modeling identified two recovery trajectories: 95.4% of patients (trajectory 1 [T1]) demonstrated steady improvement, while 4.6% (trajectory 2 [T2]) began with lower KOOS-JR scores (mean 9.7 vs. 47.9 for T1) but recovered to near T1 levels by 1 month. Trajectory 2 patients were markedly younger (mean 64 vs. 67 years), had higher Body Mass Index (36 vs. 31), included more Black or African American individuals (38% vs. 20%), and were more frequently discharged to rehabilitation facilities (16% vs. 3.3%; all *P* < 0.05). Each additional year of age reduced the likelihood of following T2 by 4% (odds ratio = 0.96, 95% confidence interval, 0.92 to 0.99; *P* = 0.016), while discharge to rehabilitation increased the likelihood 6-fold (odds ratio = 6.22, 95% confidence interval, 1.89 to 17.8; *P* = 0.001).

**Conclusion:**

This study identified two distinct recovery trajectories after TKA, with notably no trajectory emerging showing decline or stagnation from preoperative levels. Despite lower baseline scores, patients in T2 achieved substantial recovery, suggesting TKA provides meaningful improvement even for those with substantially compromised function. The findings also highlight the need to explore whether rehabilitation discharge directly influences the observed postoperative gains.

Patient-reported outcome measures have become essential tools in evaluating the success of total knee arthroplasty (TKA) from the patient's perspective. Patient-reported outcome measures are questionnaires that provide quantifiable insights into patients' pain, functional status, and overall quality of life, offering a comprehensive understanding of recovery beyond clinical and radiographic assessments.^1^ Despite the high volume of annual TKA procedures—recent US estimates project as many as 1.28 million primary TKAs annually in the United States by 2030,^2^ a notable subset of patients remains dissatisfied, with dissatisfaction rates ranging from 10% to 20%.^3-5^ Notably, some studies have further reported that a subset of patients experience no functional improvement or even a decline postoperatively, raising concerns about the benefits of the procedure for certain individuals.^6,7^

Various preoperative and postoperative factors can influence patient satisfaction after TKA. Preoperative considerations include pain severity, baseline function, and mental health, while postoperative variables encompass pain relief, functional outcomes, and knee range of motion.^4^ In addition, demographic factors such as age, race, and Body Mass Index (BMI) have been found to correlate with varying levels of postoperative outcomes, influencing patient recovery trajectories and satisfaction.^8-10^ Although some of these variables are nonmodifiable, it is important to recognize patients in the preoperative period who may be at higher risk of suboptimal recovery to provide appropriate counseling and manage their expectations.

Traditional analyses often rely on average-based or cross-sectional methods, potentially overlooking groups of patients who share distinct longitudinal recovery patterns. Growth mixture modeling (GMM) addresses this limitation by identifying latent (hidden) subgroups of individuals who share similar trajectories over time, thereby revealing systemic features that shape these patterns. GMM thus enables the detection of organically formed clusters of patients whose clinical courses may be influenced by common factors—offering opportunities to systematically target and improve outcomes within these groups. This approach is inherently exploratory because it does not presuppose the number or nature of such subgroups or trajectories, but instead allows them to emerge from the data itself.

Accordingly, the primary aim of this study was to use GMM with the Knee Injury and Osteoarthritis Outcome Score for Joint Replacement (KOOS-JR) to investigate whether there are latent recovery trajectories after TKA that represent distinctive clinical patterns. We sought to determine whether some patients experience suboptimal recovery—where the benefits of surgery may be limited—and to identify any demographic or clinical characteristics associated with these hidden trajectories. Insights from this analysis could inform how we systematically address the needs of specific patient subgroups, ultimately improving patient-centered outcomes after TKA.

## Methods

### Study Design and Setting

This retrospective exploratory cohort study was conducted at a tertiary academic center and included all patients who underwent primary, elective TKA for osteoarthritis between January 2020 and March 2023. Patients were followed for a minimum of 12 months postoperatively. Eligibility criteria included completion of a preoperative KOOS-JR questionnaire and at least two postoperative follow-up surveys. Bilateral and revision procedures were excluded. Routine KOOS-JR questionnaires were filled by patients and collected preoperatively (within 182 days before surgery) and postoperatively at 1 month (0 to 45 days), 3 months (45 to 135 days), 6 months (135 to 225 days), and 12 months (226 to 456 days). Patient characteristics collected included sex, race, age, BMI, American Society of Anesthesiologists score, marital status, smoking status, procedure type (conventional, navigation-assisted, or robotic-assisted), anesthesia type, length of stay, and discharge disposition.

### Variables, Outcome Measures, Data Sources, and Bias

The primary outcome was KOOS-JR score, ranging from 0 (complete knee disability) to 100 (perfect knee health).^11^ K-Nearest Neighbors imputation handled missing demographic and clinical data. Patients were grouped into responders (minimum of baseline survey + 2 follow-ups), incomplete responders (fewer than two follow-up surveys or no baseline survey), and nonparticipants (no KOOS-JR responses). Comparative analysis was done across these groups to address potential attrition and selection bias.

### Data Analyses

GMM estimated recovery trajectories from KOOS-JR scores, starting with a one-class solution and iterating up to six classes, testing different slope formulations. To address missing KOOS-JR data, the full-information maximum likelihood algorithm was applied, allowing trajectory estimation for the full-set of patients even when some survey time points were missing.^12^

Model selection was guided by several fit indices and diagnostic statistics, including the Akaike Information Criterion (AIC), Bayesian Information Criterion (BIC), and Vuong-Lo-Mendell-Rubin–adjusted likelihood ratio test (VLMR-LRT). Lower AIC and BIC values indicated superior model fit. The VLMR-LRT was used to compare models with k classes against those with k-1 classes, with a significant *P*-value (*P* < 0.05) indicating superior fit of the k-class model.^13^ Posterior probabilities close to 1.0, and above 0.8, reflected reliable group classification.^14^ Entropy values, ranging from 0 to 1, were used to assess class separation, with higher values representing stronger separation between classes.^15^ Log-likelihood replication and 12,000 random starts for all analysis ensured convergence and avoidance of local maxima.

Since GMM is sensitive to normality assumptions,^16^ skewness and kurtosis were assessed. When normality was violated, models were rerun using a “Skew T” distribution on high-performance computing clusters with 64-CPU virtual machines.^16^ However, these models failed to converge, a common issue reported in the literature.^17^ As a result, modelling was resumed under the assumption of normality.

Comparative statistical analyses were conducted to evaluate the demographic and clinical characteristics between the identified trajectories from GMM modeling. These included the Wilcoxon rank sum test for nonparametric data, Pearson chi-squared test for categorical variables, and Fisher exact test where appropriate.

To identify variables associated with membership in nonstandard trajectories, a “three-step approach” conforming to the “most likely class regression” methodology was used.^16^ Initially, GMM was used to model patient scores over time, identifying latent trajectory groups. Participants were then assigned to a trajectory group based on their highest posterior probability of membership. Subsequently, logistic regression was done with the trajectory classes as the outcome variables and patient characteristics as predictor variables. Variable selection for the logistic regression was conducted using a bidirectional stepwise AIC approach, which involves both forward inclusion and backward elimination of variables. This method optimizes the selection of notable variables, enhancing the model's accuracy and interpretability. All data handling and regression models were conducted using R (version 4.2.2), and Mplus 8.8 was used for GMM analyses.^18^

## Results

**Figure 1:**
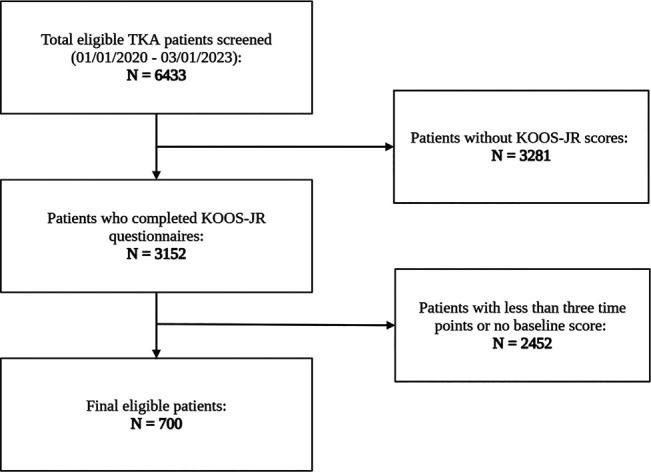
Flowchart illustrating how the study population was derived.

Of 6,433 patients, 700 patients met the inclusion criteria (Figure 1). The cohort had a median age of 67 years (IQR: 61 to 73), with a median BMI of 31.4 (IQR: 27.8 to 35.8), and 68% of participants were women. Data completeness was high, with minimal missing values for marital status (1.4%), BMI (0.7%), and smoking status (0.4%). Baseline demographics and patient characteristics are detailed in Table 1.

**Table 1:** Baseline Patient Characteristics for the Total Knee Arthroplasty Study Cohort (N = 700)

Characteristic	Median (IQR) or n (%)^a^
Procedure type	
Conventional arthroplasty knee	333 (47.6)
Navigation knee	198 (28.3)
Robotic surgery knee	169 (24.1)
Age at surgery	67 (61, 73)
Sex	
Women	475 (67.9)
Men	225 (32.1)
BMI	31 (28, 36)
Race	
White	412 (58.9)
Black or African American	147 (21.0)
Asian	46 (6.6)
Hispanic or Latino	8 (1.1)
Other	87 (12.4)
Marital status	
Currently partnered	406 (58.0)
Previously married	77 (11.0)
Single	147 (21.0)
Widowed	70 (10.0)
ASA category	
ASA 1	17 (2.4)
ASA 2	421 (60.1)
ASA 3	250 (35.7)
ASA 4	12 (1.7)
Smoking status	
Never smoker	437 (62.0)
Current smoker	22 (3.2)
Former smoker	241 (35.0)
Length of postoperative stay (hrs)	31 (27, 53)
Discharge disposition	
Home	673 (96.1)
Rehabilitation and therapy services	27 (3.9)
KOOS-JR scores	
Preoperative (baseline)	47 (37, 57)
1-month postop	55 (50, 64)
3-month postop	64 (54, 71)
6-month postop	62 (52, 68)
12-month postop	62 (52, 72)

The bolded row is the final model utilized in this study and the final analysis.

ASA = American Society of Anesthesiologists, BMI = Body Mass Index, IQR = interquartile range, KOOS-JR = Knee Injury and Osteoarthritis Outcome Score for Joint Replacement, n = number of patients

a Data are presented as median (IQR) for continuous variables and n (%) for categorical variables.

This table includes imputed values for missing data: marital status (1.4%, n = 10), BMI (0.7%, n = 5), and smoking status (0.4%, n = 3).

### Latent Recovery Trajectories

**Figure 2:**
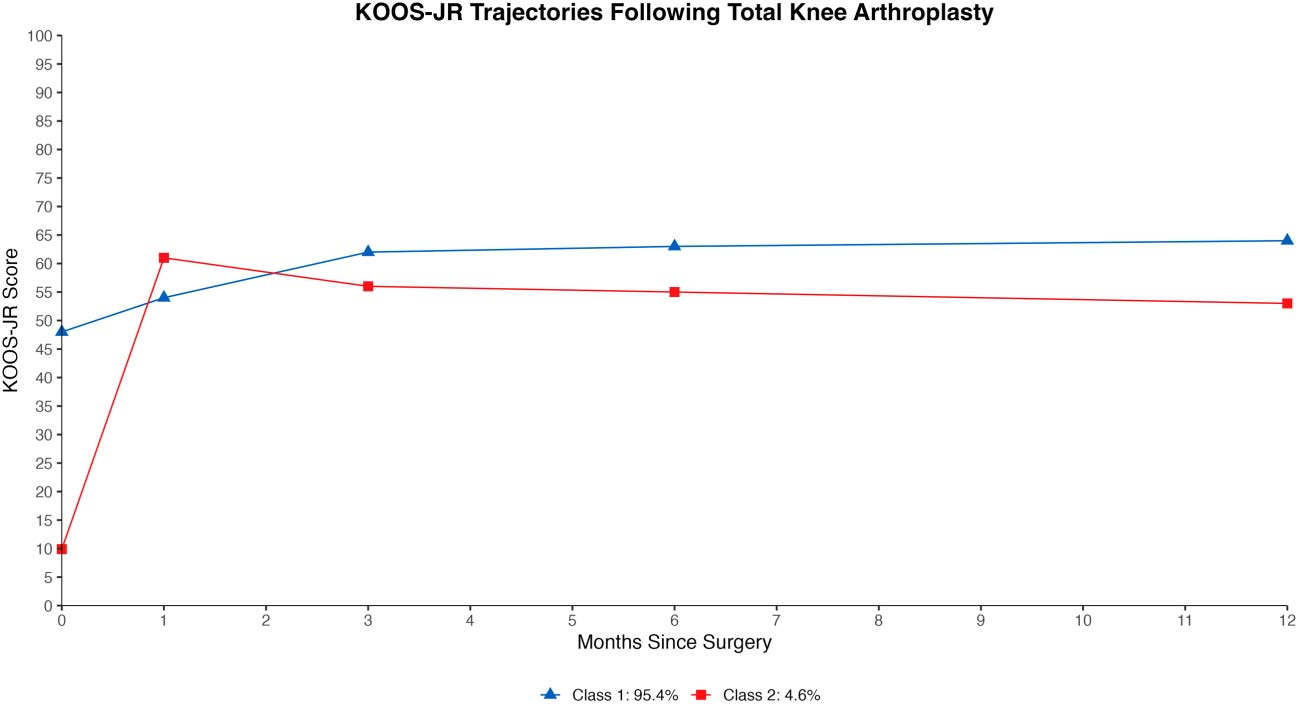
Piecewise two-class growth mixture model projecting latent trajectories in patient KOOS-JR scores over the first postoperative year after total knee arthroplasty (TKA). KOOS-JR = Knee Injury and Osteoarthritis Outcome Score for Joint Replacement, T1 = trajectory 1, T2 = trajectory 2

GMM identified two distinct latent recovery trajectories based on KOOS-JR scores (Figure 2). The final model was chosen because of demonstrating excellent fit, with a significant VLMR-LRT (*P* < 0.001), the lowest BIC, and high entropy (0.93), indicating strong classification accuracy. Posterior probabilities further supported classification accuracy, with a minimum probability of 0.86 (Table 2).

**Table 2:** Goodness-of-Fit Parameters of all Growth Mixture Models Generated for the Trajectories

Model	Classes	AIC	BIC	Entropy	Minimum PP	Minimum class size (%)	VLMR-LRT *P*
Linear Quadratic Piecewise (2 × 4)	1 1 1	21,166.50 21,035.70 20,897.11	21,212.03 21,099.43 20,965.40	— — —	— — —	— — —	— — —
Linear Quadratic Piecewise (2 × 4)	2 2 **2**	21,124.50 20,996.94 **20,836.31**	21,183.68 21,078.89 **20,927.36**	0.977 0.961 **0.927**	0.921 0.822 **0.861**	1.0 1.9 **4.6**	0.0308 0.0682 **0.0057**
Linear Quadratic Piecewise	3 3 3	21,121.50 20,989.22 20,775.722	21,194.34 21,089.37 20,889.53	0.948 0.969 0.939	0.782 0.838 0.869	0.3 0.1 1.4	0.2653 0.0165 0.0196
Linear Quadratic Piecewise (2 × 4)	4 4 4	21,109.97 20,980.69 20,751.56	21,196.47 21,099.06 20,888.14	0.694 0.910 0.946	0.755 0.757 0.882	0.4 0.3 0.9	0.2119 0.0658 0.1720
Linear Quadratic Piecewise (2 × 4)	5 5 5	21,105.74 20,971.39 20,743.56	21,205.89 21,107.97 20,902.90	0.729 0.921 0.757	0.686 0.751 0.802	0.3 0.1 0.9	0.3597 0.0004 0.5151
Linear Quadratic Piecewise (2 × 4)	6 6 6	21,098.70 20,966.68 20,734.73	21,212.52 21,121.47 20,916.83	0.745 0.805 0.782	0.707 0.741 0.799	0.3 0.3 0.3	0.0140 0.5059 0.2008

The bolded enteries are where *p* value is less than 0.05 i.e suggesting significance.

2 × 4 = model where the baseline to 1-month period is one segment, and the remaining time points form the second segment, AIC = Akaike Information Criterion, BIC = Bayesian Information Criterion, PP = posterior probability = VLMR-LRT *P*-value, *P*-values from the Vuong-Lo-Mendell-Rubin likelihood ratio test

Trajectory 1 (T1) represented most of the patients (95.4%, n = 668). These patients had moderate preoperative KOOS-JR scores, with a mean of 47.9, and experienced gradual, steady improvement throughout the postoperative period. At the 1-month follow-up, the mean KOOS-JR score for T1 had increased to 54.6, and additional gains were observed over time, stabilizing at a mean score of 63.3 by 12 months. This trajectory was considered the standard recovery pathway for most patients.

By contrast, trajectory 2 (T2) comprised a smaller subgroup (4.6%, n = 32) who entered surgery with severely impaired knee function, reflected in a mean preoperative KOOS-JR score of 9.7. Patients in this group experienced substantial improvement within the first month after surgery, reaching a mean KOOS-JR score of 61.0, surpassing the 1-month score of T1 patients. However, after this rapid early gain, their scores slightly declined and then stabilized at a mean of 52.4 by the 12-month follow-up.

### Demographic and Clinical Characteristics of Trajectories

Significant demographic and clinical differences were observed between the two recovery trajectories (Table 3). Patients in trajectory T2 were younger, with a median age of 64 years (IQR: 58 to 68), compared with 67 years (IQR: 61 to 73) in trajectory T1 (*P* = 0.035). Trajectory 2 patients also had a higher median BMI of 36 (IQR: 31 to 40) compared with 31 (IQR: 28 to 36) in T1 (*P* = 0.007). A greater proportion of patients in T2 identified as Black or African American (38 vs. 20%; *P* = 0.016). In addition, T2 patients were more frequently discharged to rehabilitation or therapy services (16%) compared with those in T1 (3.3%; *P* = 0.006).

**Table 3:** Comparative Statistics Between Patients Allocated to Latent Trajectories T1 and T2, Identified Through Growth Mixture Modelling (N = 700)

Characteristic	Trajectory Group 1, N = 668^a^	Trajectory Group 2, N = 32^a^	*P* ^b^
Procedure type			0.119
Conventional arthroplasty knee	316 (47.0%)	17 (53.1%)	
Navigation knee	186 (28.0%)	12 (37.5%)	
Robotic surgery knee	166 (25.0%)	3 (9.4%)	
Age at surgery	67 (61, 73)	64 (58, 68)	**0.035**
Sex			
Women	451 (67.5%)	24 (75.0%)	
Men	217 (32.5%)	8 (25.0%)	
BMI	31 (28, 36)	36 (31, 40)	**0.007**
Race			**0.016**
White	399 (59.7%)	13 (40.6%)	
Black or African American	135 (20.2%)	12 (37.5%)	
Asian	44 (6.6%)	2 (6.2%)	
Hispanic or Latino	6 (0.9%)	2 (6.2%)	
Other	84 (12.6%)	3 (9.5%)	
Marital status			0.331
Currently partnered	392 (58.8%)	16 (50.0%)	
Single or previously married	207 (31.0%)	14 (43.8%)	
Widowed	69 (10.2%)	2 (6.2%)	
ASA category			0.697
ASA 1	17 (2.6%)	0 (0.0%)	
ASA 2	402 (60.1%)	19 (59.4%)	
ASA 3	238 (35.6%)	12 (37.5%)	
ASA 4	11 (1.7%)	1 (3.1%)	
Smoking status			0.159
Never smoker	414 (62.0%)	23 (71.9%)	
Current smoker	20 (3.0%)	2 (6.20%)	
Former smoker	234 (35.0%)	7 (21.9%)	
Postop length of stay (hrs)	31 (27, 52)	33 (30, 54)	0.229
Discharge disposition			**0.006**
Home	646 (96.7%)	27 (84.4%)	
Rehabilitation and therapy services	22 (3.3%)	5 (15.6%)	

The bolded enteries are where *p* value is less than 0.05 i.e suggesting significance.

ASA = American Society of Anesthesiologists, BMI = Body Mass Index, IQR = interquartile range

a Data are presented as median (IQR) for continuous variables and n (%) for categorical variables.

b Wilcoxon rank sum test; Pearson chi-squared test; Fisher exact test.

### Factors Associated With Trajectory Group Membership

Logistic regression analysis revealed that both age and discharge to rehabilitation were key factors associated with membership to the T2 trajectory group (Table 4). Older patients were less likely to follow T2, with each additional year of age reducing the odds of being in this trajectory by 4% (odds ratio = 0.96, 95% confidence interval, 0.92 to 0.99; *P* = 0.016). Conversely, patients discharged to rehabilitation or therapy services had a 6-fold increase in the likelihood of following the T2 trajectory (OR = 6.22, 95% confidence interval, 1.89 to 17.8; *P* = 0.001).

**Table 4:** Logistic Regression Results Indicating Odds Ratios for Allocation to Trajectory T2 vs. Standard Trajectory T1, After Stepwise Bidirectional Variable Selection Based on the Akaike Information Criterion

Characteristic	Trajectory 2		
	OR	95% CI	*P*
Procedure type			
Conventional arthroplasty knee	—	—	—
Navigation knee	1.25	0.56-2.73	0.571
Robotic surgery knee	0.36	0.08-1.11	0.111
Age at surgery	0.96	0.92-0.99	**0.016**
Discharge disposition			
Home	—	—	—
Rehabilitation and therapy services	6.22	1.89-17.80	**0.001**

The bolded enteries are where *p* value is less than 0.05 i.e suggesting significance.

CI = confidence interval, OR = odds ratio

### Response Rates and Patient Characteristics

Among the 6,433 patients who underwent a primary elective TKA due to osteoarthritis in the study period, 700 patients (10.9%) were classified as responders, 2,452 (38.1%) as incomplete responders, and 3,281 (51.0%) as nonparticipants. Of the responders, 100% had preoperative KOOS-JR scores, 96.14% (n = 673) had 1-month scores, 59.86% (n = 419) had 3-month scores, 65.42% (n = 458) had 6-month scores, and 47.28% (n = 331) had 12-month scores.

Significant differences were noted between groups (Table 5). Conventional TKA procedures were most common among nonparticipants (74%), followed by incomplete responders (53%) and responders (48%; *P* < 0.001). Conversely, navigation-assisted and robotic-assisted procedures were more frequent among responders (28 and 24%, respectively) compared with incomplete responders (25 and 22%) and nonparticipants (13% for both). In addition, there were significant differences in racial distribution (*P* < 0.001) and marital status (*P* = 0.001), with fewer partnered individuals among nonparticipants (55%) compared with responders (58%) and incomplete responders (59%).

**Table 5:** Patient Characteristics for Responders, Incomplete Responders, and Nonparticipants in the Knee Injury and Osteoarthritis Outcome Score for Joint Replacement Questionnaire (N = 6,433)

Characteristic	Incomplete Responders, N = 2,452^a^	Nonparticipants, N = 3,281^a^	Responders, N = 700^a^	*P* ^b^
Procedure type				<0.001
Conventional arthroplasty knee	1,291 (53.0%)	2415 (74.0%)	333 (48.0%)	
Navigation knee	620 (25.0%)	424 (13.0%)	198 (28.0%)	
Robotic surgery knee	541 (22.0%)	442 (13.0%)	169 (24.0%)	
Age at surgery	67 (61, 73)	68 (61, 74)	67 (61, 73)	0.056
Sex				0.619
Women	1616 (66.0%)	2183 (67.0%)	475 (68.0%)	
Men	836 (34.0%)	1098 (33.0%)	225 (32.0%)	
BMI	31.5 (27.8, 35.8)	31.8 (28.1, 36.0)	31.2 (27.7, 36.4)	0.216
Race				**<0.001**
White	1338 (58.4%)	1616 (50.8%)	412 (58.9%)	
Black or African American	514 (22.4%)	757 (23.8%)	147 (21.0%)	
Asian	77 (3.4%)	116 (3.6%)	46 (6.6%)	
Hispanic or Latino	65 (2.8%)	314 (9.9%)	8 (1.1%)	
Other	299 (13.0%)	378 (11.9%)	87 (12.4%)	
Marital status				**0.001**
Currently partnered	1429 (59.0%)	1761 (55.0%)	399 (57.9%)	
Previously married	244 (10.0%)	338 (10.0%)	76 (11.1%)	
Single	481 (20.0%)	668 (21.0%)	145 (21.0%)	
Widowed	260 (11.0%)	456 (14.0%)	79 (10.0%)	
ASA category				**<0.001**
ASA 1	71 (2.9%)	69 (2.1%)	17 (2.4%)	
ASA 2	1406 (57.0%)	1755 (53.0%)	421 (60.1%)	
ASA 3	957 (39.0%)	1413 (43.0%)	250 (35.7%)	
ASA 4	18 (0.7%)	44 (1.3%)	12 (1.7%)	
Smoking status				**0.034**
Never smoker	1482 (61.0%)	2018 (62.0%)	435 (62.4%)	
Current smoker	95 (3.9%)	168 (5.2%)	22 (3.2%)	
Former smoker	853 (35.0%)	1067 (33.0%)	240 (34.4%)	
Postop length of stay (hrs)	31 (27, 52)	33 (29, 55)	31 (27, 53)	**<0.001**
Discharge disposition				**<0.001**
Home	2,309 (94.0%)	2,990 (91.0%)	673 (96%)	
Rehabilitation and therapy services	143 (5.8%)	291 (8.9%)	27 (3.9%)	

The bolded enteries are where *p* value is less than 0.05 i.e suggesting significance.

ASA = American Society of Anesthesiologists, BMI = Body Mass Index, KOOS-JR = Knee Injury and Osteoarthritis Outcome Score for Joint Replacement, IQR = interquartile range

a n (%); Median (IQR).

b Pearson chi-squared tests; Kruskal-Wallis rank sum test.

There are missing values for race among incomplete responders (6.5%, n = 159) and nonparticipants (3.0%, n = 100). Marital status also has incomplete data for incomplete responders (1.6%, n = 38), nonparticipants (1.8%, n = 58), and responders (1.4%, n = 10). Smoking status shows missing data for incomplete responders (0.9%, n = 22), nonparticipants (0.9%, n = 28), and responders (0.4%, n = 3). BMI data are missing for responders (0.7%, n = 5). No imputation was done on this table.

## Discussion

This study identified two distinct recovery trajectories after TKA and their associated characteristics. Patients achieved most recovery by 3 months, regardless of the trajectory. Most of the patients exhibited typical and anticipated recovery patterns with sustained improvement after their TKA; however, there was a small subset of patients who presented with very low preoperative scores. Notably, growth mixture modelling did not discern the presence of any trajectory that demonstrates a pattern of worsening or stagnation in knee function, suggesting that there is unlikely to be a systemic group of patients who fail to benefit postoperatively.

These findings align with and build on the existing literature. Hamilton et al^19^ identified three recovery trajectories (poor, moderate, and good responders), with most patients being good responders. Similar to our results, their study noted improvements up to 3 months postoperatively, with recovery plateauing by 6 months. Christensen et al^20^ also identified a single trajectory with substantial functional improvement within the first 3 months. These consistent findings across studies highlight the importance of the early postoperative period, during which targeted interventions may have the greatest effect on patient outcomes.

However, in contrast to previous research which identified subgroups of patients with minimal or no improvement after TKA,^6,7^ this study did not reveal any trajectories of worsening or stagnation. This suggests that patients with suboptimal outcomes may not follow sufficiently homogeneous recovery patterns to form a distinct subgroup, indicating that individualized factors may play a larger role in such cases. This observation presents a need for a case-by-case approach in managing patients with slower or more complicated recoveries, rather than assuming they represent a broader, easily identifiable group.

Our findings further demonstrated that demographic and clinical factors markedly influenced the recovery trajectories. Patients in the T2, who had poor preoperative function, were generally younger, had higher BMIs, and included a greater proportion of African American patients. The high proportion of Black or African American patients in T2 aligns with studies showing that these patients often report lower preoperative scores, but no notable postoperative differences, possibly due to factors such as pain catastrophizing, healthcare disparities, and differences in physical and mental health.^9,21-23^ Similarly, our findings regarding BMI are supported by the literature because higher BMI has been consistently associated with poorer preoperative function but greater relative postoperative improvement.^8,24^

Interestingly, although patients in T2 had a higher prevalence of elevated BMI, it was not independently associated with trajectory membership in the logistic regression analysis after adjusting for other variables. This discrepancy may be due to the relationship between increased BMI and discharge to rehabilitation facilities, as documented in previous studies.^25-27^ Keeney et al^28^ found that morbidly obese patients who did not lose substantial weight before surgery were more likely to be discharged to rehabilitation facilities. This suggests that although BMI may not directly predict recovery trajectories, its influence on postoperative care requirements and discharge planning may play a more critical role in shaping recovery outcomes.

In addition, older age decreased the likelihood of following the T2 trajectory. This finding is consistent with previous research indicating that younger patients, often with higher preoperative pain levels, tend to exhibit more rapid postoperative improvements.^10,29^ However, although younger patients may engage more intensively in rehabilitation, our study provides no evidence suggesting older patients derive less benefit from TKA. In fact, older individuals were more likely to follow a typical recovery trajectory, suggesting that older age is not a barrier to achieving successful outcomes.

Our study also found that discharge to rehabilitation facilities was strongly associated with membership to the T2 trajectory. Since this factor only comes into play postoperatively, it suggests that rehabilitation may contribute to the rapid recovery seen. However, this association should be interpreted with caution because the utility and benefit of facilities have been widely debated in the literature and are unlikely to be purely causative.^27,30-32^ It is possible that patients with worse preoperative conditions have a greater need for rehabilitation, which increases their likelihood of being discharged to these facilities, rather than the facilities themselves driving their recovery. Additional research is needed to disentangle these effects and clarify the role that rehabilitation facilities play in postoperative recovery.

This study provides novel insights through the application of GMM to better understand recovery patterns after TKA. Whereas prior approaches have focused on individual patient factors and outcomes,^10,33^ GMM identifies latent subgroups of patients with similar trajectories, yielding associations that are more meaningful for larger populations. This method allows recovery patterns to emerge organically from the data, providing an unbiased perspective.

The ability to analyze recovery as a group-based phenomenon presents clear clinical benefits. Identifying and categorizing patients based on their recovery trajectories provides actionable insights that are more broadly applicable. For instance, patients with low preoperative function who show rapid improvement postoperatively may require tailored interventions that differ from those for patients on a more standard recovery path. Targeting interventions in this way is not only clinically effective but may also be more cost-efficient. Since these interventions are designed to address the needs of entire patient subgroups with similar recovery profiles, rather than customizing care for each individual, healthcare systems can allocate resources more efficiently, ultimately benefiting larger numbers of patients.

This group-based approach also enhances the preoperative counseling process. Based on knowledge of trajectories, surgeons can provide more personalized guidance, setting realistic expectations and better preparing patients for the types of interventions and monitoring they may require throughout their recovery.

## Limitations

This study has several limitations. A key concern is attrition bias: only 10.9% of patients met our strict criteria for “responders,” while over half (51.0%) were classified as nonparticipants. These stringent criteria were chosen to maximize the granularity of trajectory data. Although simpler criteria requiring fewer completed surveys might have yielded a higher responder rate, capturing detailed postoperative trajectories was ultimately prioritized.

In addition, nonparticipants were more likely to have undergone conventional TKA, potentially introducing selection bias. Unmeasured factors such as socioeconomic status, education level, and patient-surgeon relationships may also have influenced response rates. Furthermore, response bias is evident in the overrepresentation of Asian patients and underrepresentation of Hispanic or Latino patients, suggesting possible disparities in healthcare access or cultural differences in survey participation.

Moreover, owing to the retrospective nature of this study, we lacked data on mental health scores and knee range of motion, which may have been notable predictors of recovery trajectories. However, since predictors do not define the trajectories themselves, the absence of these variables limits our ability to assess their association with the trajectories identified, rather than altering their underlying patterns.

Generalizability may be limited by the study's single-center, urban setting. Although the overall sample size was sufficient to detect major differences in recovery trajectories, the small number of T2 patients could reduce the power to identify subtler associations within this subgroup. Larger, multicenter studies are needed to validate these findings in more diverse populations.

Finally, this study was conducted during the COVID-19 pandemic, which—despite not markedly altering TKA practices at our institution—may have affected postoperative care, particularly in rehabilitation facilities. The pandemic could also have contributed to lower follow-up response rates, a factor that should be taken into account when interpreting the results.

## Conclusion

This study identified two distinct recovery trajectories after TKA, with most patients showing notable improvement within 3 months. There was no evidence of a distinct subgroup exhibiting consistently stagnant or worsening function, suggesting that systemic failure after TKA is unlikely. Patients with severely low preoperative function also demonstrated substantial recovery and were more frequently discharged to rehabilitation facilities. These findings reinforce the notion that even patients with severely poor preoperative function can expect meaningful recovery. Additional research is needed to determine whether rehabilitation directly influences these improvements or simply reflects the complexity of their care needs. Clarifying this relationship could help reshape the role of rehabilitation facilities in optimizing postoperative outcomes for TKA patients.
